# Supplementary material for: Characterization of Human Immunodeficiency Virus-1 Transmission Clusters and Transmitted Drug-Resistant Mutations in Croatia from 2019 to 2022
Source: Viruses. 2023 Dec 11;15(12):2408. doi: 10.3390/v15122408 (PMC10747707; doi:10.3390/v15122408)
Supplement: Supplementary file 1 [file viruses-15-02408-s001.zip › viruses-2750133-supplementary.pdf]

Characterization of HIV-1 transmission clusters and transmitted drug-resistant mutations in Croatia from 2019 to 2022

Ana Planinić[1], Josip Begovac[1,2], Filip Rokić[3], Petra Šimičić[4], Maja Oroz[5], Katja Jakovac[3], Oliver Vugrek[3] and Snježana Židovec Lepej[1]

[1] Department of Immunological and Molecular Diagnostics, University Hospital for Infectious Diseases "Dr. Fran Mihaljević", 10000 Zagreb, Croatia; anaplaninic@yahoo.com (A.P.), szidovec@gmail.com (S.Z.L.)

[2] University of Zagreb School of Medicine, 10000 Zagreb, Croatia; josip.begovac@gmail.com (J.B.)

[3] Ruđer Bošković Institute, 10000 Zagreb, Croatia; frokic@irb.hr (F.R); oliver.vugrek@irb.hr (O.V.), katjajakovac@gmail.com (K.J.)

[4]Department of Oncology and Nuclear Medicine, Sestre milosrdnice University Hospital Center, 10000 Zagreb, Croatia; petrasimicic@gmail.com (P.S.)

[5]Cytogenetic Laboratory, Department of Obstetrics and Gynecology, Clinical Hospital "Sveti Duh", Zagreb, Croatia: majatrupkovic@gmail.com (M.O.)

Correspondence: szidovec@gmail.com (S.Z.L.)

Table S1. Primers for PCR and Sanger sequencing (SS) of HIV-1 PR/RT and IN region

**Primers for PCR- RT/PI region**	**Sequence (5'->3')**
JA269	AGGAAGGACACCARATGA ARG A
JA272	GGATAAATCTGACTTGCCCAR T
JA270	GCTTCCCTCARATCACTCTT
JA271	CCACTAAYTTCTGTATRTCATTGAC
**Primers for PCR- INT region**	**Sequence (5'->3')**
HIV-1-INT-O-F	GAGTTTGTCAATACCCCTCCC
HIV-1-INT-O-R	CCTTGTTATGTCCTGCTTGATATTCAC
HIV-1-INT-N-F	GGAATTGGAGGAAATGAACAAG
HIV-1-INT-N-R	CCCTAGTGGGATGTGTAC
**Primers for SS-RT/PI region**	**Sequence (5'->3')**
JA273	CCCTCARATCACTCTTTGGC
JA276	TGTATRTCATTGACAGTCCA
JA305	ATTCCTAATTGRACYTCCCA
JA311	AAAATCCATAYAAYACTCCA
**Primers for SS-INT region**	**Sequence (5'->3')**
HIV-1-INT-N-F	GGAATTGGAGGAAATGAACAAG
HIV-1-INT-seq-F2	TTATCCTGGTAGCAGTTCATGTA
HIV-1-INT-seq-R3	ACTGTACCCCCCAATCCCC
HIV-1-INT-N-R	CCCTAGTGGGATGTGTAC

PR- protease; RT-reverse transcriptase; IN-integrase

Table S2. Primers for deep sequencing library of HIV-1 protease (codons 1-99), reverse transcriptase (codons 1-400) and integrase (codons 1-288) region.

**Primers for HIV-1 PR/RT region**	**FW primer, sequence (5'->3')**	**RV primer, sequence (5'->3')**
Primer 1	AGCCGATAGACAAGGAACTG	GATAAAACCTCCAATTCCCCCTAT
Primer 2	GCTCTATTAGATACAGGAGCAGAT	CAATTATGTTGACAGGTGTAGGT
Primer 3	GAAATCTGTGGACATAAAGCTATAGG	GGCCATTGTTTAACTTTTGGG
Primer 4	TAGCCCTATTGAGACTGTACCAG	ACTGGAGTATTGTATGGATTTTCAG
Primer 5	GTACAGAGATGGAAAAGGAAGG	CGGGATGTGGTATTCCTAATTG
Primer 6	GAGAACTCAAGACTTCTGGGAAG	GTACTGATATCTAATCCCTGGTGTC
Primer 7	ACAGTACTGGATGTGGGTGATG	AGGCTCTAAGATTTTTGTCATGCTAC
Primer 8	TGTGCTTCCACAGGGATG	AGATGTTGTCTCAGCTCCTC
Primer 9	GGATCTGACTTAGAAATAGGGC	AGCACTATAGGCTGTACTGTC
Primer 10	CATCAGAAAGAACCTCCATTCCTT	CTTTAATCCCTGGGTAAATCTGACT
Primer 11	CAATGACATACAGAAGTTAGTGG	GGTCATAATACACTCCATGTACTG
Primer 12	CACTAACAGAAGAAGCAGAGCTAG	CCCTCATTCTTGCATATTTTCCTGT
Primer 13	GGACATATCAAATTTATCAAGAGCC	ACCATGTTTCCCATGTTTCC
**Primers for HIV-1 IN region**	**FW primer, sequence (5'->3')**	**RV primer, sequence (5'->3')**
Primer 14	GAATTGGAGGAAATGAACAAGTAG	GCTGACATTTATCACAGCTGG
Primer 15	GGCTAGTGATTTTAACCTGCC	CACTGGCTACATGAACTGCT
Primer 16	GCATGGACAAGTAGACTGTAGT	GTTTTTAGGCCATCTTCCTGC
Primer 17	GAAACAGGGCAGGAAACAGC	CTTGACTTTGGGGATTGTAGG
Primer 18	CAGCAATTTCACCGGTGCT	GATGAATACTGCCATTTGTACTGC
Primer 19	GGTAAGAGATCAGGCTGAACAT	GCTGTCCCTGTAATAAACCCG
Primer 20	GGGGAAAGAATAGTAGACATAATAGCAAC	GTCACTATTATCTTGTATTACTACTGCCC
Primer 21	AAGCTCCTCTGGAAAGGTGAAG	CCATGTTCTAATCCTCATCCTGT
Primer 22	TGCCAAGAAGAAAAGCAAAGAT	TGAGGGCTTTCATAGTGATGT

	C	

PI- protease; RT-reverse transcriptase; IN-integrase; FW-forward; RV:-reverse

Table S3. Overall time trends for subtype distribution for study population in period 2019-2022.

HIV subtypes, n (%)	All n=270	2019	2020	2021	2022
**B**	198	67	42	49	40
**A**	12	4	3	2	3
**C**	3	/	1	1	1
**F**	3	/	/	1	2
**CRFs**					
CRF12_BF	4	2	/	/	2
CRF01_AE	4	1	1	1	1
CRF06_CPX	2	1	/	1	/
Other CRFs	3	1	/	1	1
**Recombinants**					
BD	29	5	3	7	14
Other recombinants	12	2	3	4	4

CRF-circulating recombinant form

Table S4. Patterns of SDRM in newly diagnosed treatment naive persons living with HIV (PLHIV) in period 2019-2022

Risk Factor	Age	Subtype	SDRM to NRTI	SDRM to NNRTI	SDRM to PI	SDRM to InSTI	Clinical relevance according to IAS list	Clinical relevance according to Stanford HIVdb
MSM	33	B	S	L100LI, K103KN	S	S	Clinically resistant strain	Intermediate to high-level resistance
MSM	32	B	S	K101E	S	S	Clinically resistant strain	Intermediate to high-level resistance
MSM	18	B	T215D	L100I, K103N	V32I, I47V, F53L	S	Clinically resistant strain	Potential low-level resistance to high-level resistance
MSM	33	B	L210W, T215S	S	S	S	Clinically resistant strain	Low-level to intermediate resistance
MSM	42	B	T215S	S	S	S	Susceptible	Low-level resistance
HETERO	30	B	S	K101E	S	S	Clinically resistant strain	Intermediate to high-level resistance
HETERO	23	CRF12_BF	S	K103N	S	S	Clinically resistant strain	High-level resistance
MSM	37	B	L210W, T215S	S	S	S	Clinically resistant strain	Low-level to intermediate resistance
HETERO	33	B	S	K101E	S	S	Clinically resistant strain	Intermediate to high-level resistance
MSM	63	B	S	K103N	S	S	Clinically resistant strain	High-level resistance
MSM	54	B	S	K103N, P225H	S	S	Clinically resistant strain	Intermediate to high-level resistance
MSM	30	B	T215S	S	S	S	Susceptible	Low-level resistance
MSM	48	B	S	K103N	S	S	Clinically resistant strain	High-level resistance
MSM	20	B/D	K219R	S	S	S	Susceptible	Potential low-level resistance

MSM	55	B	S	K101E	S	S	Clinically resistant strain	Intermediate to high-level resistance
MSM	25	B	T215S	S	S	S	Susceptible	Low-level resistance
HETERO	32	B	S	K101E	S	S	Clinically resistant strain	Intermediate to high-level resistance
MSM	56	B	M41L, T215Y	S	S	S	Clinically resistant strain	Low-level to high level resistance
MSM	27	B	T215S	S	S	S	Susceptible	Low-level resistance
MSM	36	B	T215S	S	S	S	Susceptible	Low-level resistance
MSM	20	B	T215S	S	S	S	Susceptible	Low-level resistance
MSM	21	B	T215S	S	S	S	Susceptible	Low-level resistance
MSM	22	B	L210W, T215S	S	S	S	Clinically resistant strain	Low-level to intermediate resistance
MSM	24	B	T215S	S	S	S	Susceptible	Low-level resistance
HETERO	27	B	S	K101E	S	S	Clinically resistant strain	Intermediate to high-level resistance
MSM	46	B	T215S	S	S	S	Susceptible	Low-level resistance
MSM	33	B/D	S	S	I85V	S	Susceptible	Susceptible
MSM	57	B	T215S	S	S	S	Susceptible	Low-level resistance
MSM	31	B	T215S	S	S	S	Susceptible	Low-level resistance
MSM	53	B	S	K101E	S	S	Clinically resistant strain	Intermediate to high-level resistance
MSM	29	B/D	T215S	S	S	S	Susceptible	Low-level resistance
MSM	50	B	S	K103N	S	S	Clinically resistant strain	High-level resistance
MSM	28	B/D	T215E	L100I, K103N,	V32I, I47V, F53L	S	Clinically resistant strain	Low-level to high-level resistance
MSM	20	B	T215S	S	S	S	Susceptible	Low-level resistance
MSM	55	B	T215S	S	S	S	Susceptible	Low-level resistance
MSM	49	B	S	K101E	S	S	Clinically resistant strain	Intermediate to high-level resistance

HETERO	55	B		K101E	S	S	Clinically resistant strain	Intermediate to high-level resistance
MSM	40	CRF 39_BF	D67N, T69D, T215S, K219Q	S	D30N, N88D	S	Clinically resistant strain	Low-level to intermediate resistance
MSM	48	B	S	K103N	S	S	Clinically resistant strain	High-level resistance
MSM	38	B	T215S	S	S	S	Susceptible	Low-level resistance
MSM	42	B	L210W, T215S,	S	S	ND	Clinically resistant strain	Low-level to intermediate resistance
UKN	38	B	T215S	S	S	ND	Susceptible	Low-level resistance
MSM	30	A1	S	K103N	S	S	Clinically resistant strain	High-level resistance
MSM	33	B/D	T215S	S	S	S	Susceptible	Low-level resistance
MSM	27	B/D	S	S	I85V	S	Susceptible	Susceptible

MSM: men who have sex with men; SDRM: surveillance drug resistance mutation; NRTI: nucleoside reverse transcriptase inhibitors; NNRTI: non-nucleoside reverse transcriptase inhibitors; PI: protease inhibitors; InSTI: integrase strand-transfer inhibitors; ND: not determined

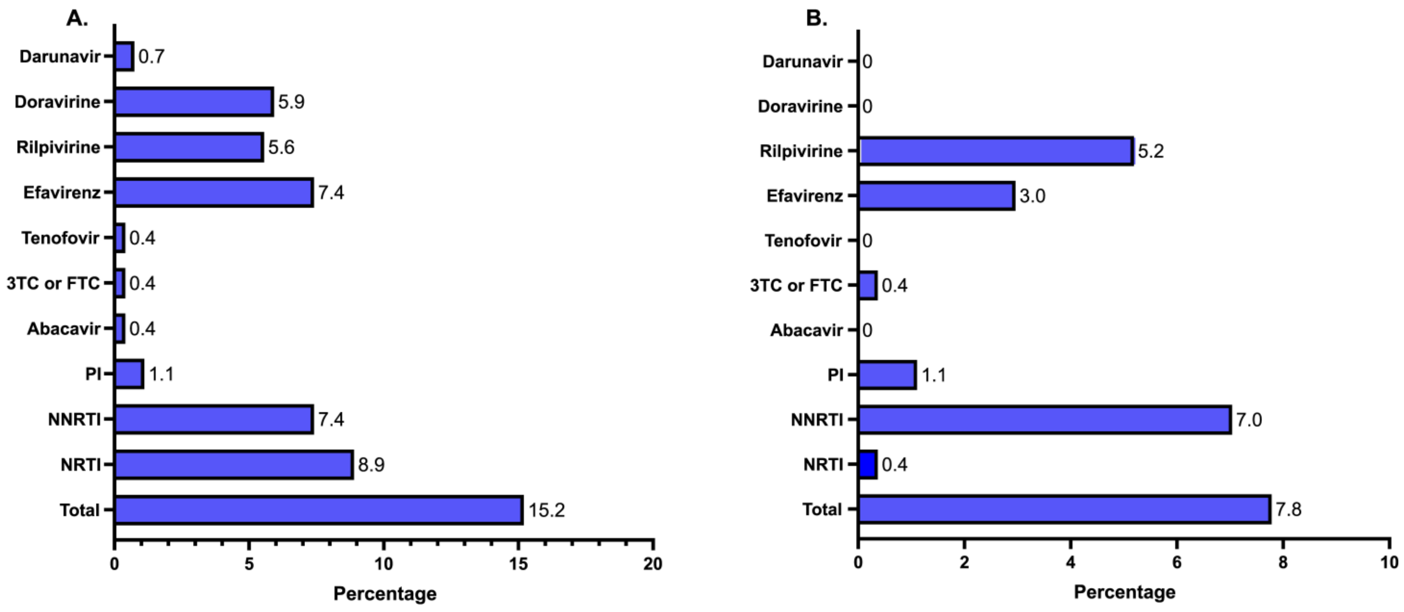

Figure S1. Prevalence of transmitted drug resistance in 270 persons entering HIV care in Croatia, 2019-2022. Presented are prevalences resulting in at least low-level resistance (Stanford score ≥15) (panel A) or high level resistance (Stanford score ≥60) (panel B) to selected antiretrovirals, antiretroviral classes, and total. NRTI-Nucleoside reverse transcriptase inhibitors. NNRTI- Non-nucleoside reverse transcriptase inhibitors. PI-protease inhibitors. 3TC-lamivudine, FTC-emtricitabine.

Table S5. Characteristics and resistance profile of PLHIV with mutations E138A and E138K

Age	Transmission risk	Subtype	All mutations (all regions)	Primary resistance	Clinical relevance according to Stanford HIVdb	Clinical relevance according to IAS list
32	MSM	B	K101E, E138K	yes	DOR (LLR)<br>EFV (LLR)<br>ETR (LLR)<br>NVP (IR)<br>RPV (HLR)	Resistance to RPV
30	HETERO	B	K101E, E138K	yes	DOR (LLR)<br>EFV (LLR)<br>ETR (LLR)<br>NVP (IR)<br>RPV (HLR)	Resistance to RPV
47	HETERO	B	V106I, E138A	no	DOR (PLLR)<br>ETR (LLR)<br>NVP (PLLR)<br>RPV (LLR)	Resistance to RPV
52	HETERO	B	V106I, E138A	no	DOR (PLLR)<br>ETR (LLR)<br>NVP (PLLR)<br>RPV (LLR)	Resistance to RPV
33	HETERO	B	K101E, E138K, V118I	yes	DOR (LLR)<br>EFV (LLR)<br>ETR (LLR)<br>NVP (IR)<br>RPV (HLR)	Resistance to RPV
43	MSM	A1	E138A	no	ETR (PLLR)<br>RPV (LLR)	Resistance to RPV
55	MSM	B	K101E, E138K, V179T	yes	DOR (LLR)<br>EFV (LLR)<br>ETR (LLR)	Resistance to RPV

					NVP (IR)<br>RPV (HLR)	
32	HETERO	B	K101E,<br>E138K	yes	DOR (LLR)<br>EFV (LLR)<br>ETR (LLR)<br>NVP (IR)<br>RPV (HLR)	Resistance to RPV
48	MSM	B	E138A	no	ETR (PLLR)<br>RPV (LLR)	Resistance to RPV
48	MSM	A1	E138A	no	ETR (PLLR)<br>RPV (LLR)	Resistance to RPV
24	MSM	A1	E138A	no	ETR (PLLR)<br>RPV (LLR)	Resistance to RPV
36	MSM	A1	E138A	no	ETR (PLLR)<br>RPV (LLR)	Resistance to RPV
27	MSM	A1	E138A	no	ETR (PLLR)<br>RPV (LLR)	Resistance to RPV
27	HETERO	B	K101E,<br>E138K	yes	DOR (LLR)<br>EFV (LLR)<br>ETR (LLR)<br>NVP (IR)<br>RPV (HLR)	Resistance to RPV
53	MSM	B	K101E,<br>V106VI,<br>E138K,<br>K103KR	yes	DOR (IR)<br>EFV (LLR)<br>ETR (IR)<br>NVP (IR)<br>RPV (HLR)	Resistance to RPV
50	MSM	B	E138A	no	ETR (PLLR)<br>RPV (LLR)	Resistance to RPV
49	MSM	B	K101E,<br>E138K	yes	DOR (LLR)<br>EFV (LLR)<br>ETR (LLR)<br>NVP (IR)<br>RPV (HLR)	Resistance to RPV

55	HETERO	B	A62AV, V75VI K101E, E138K, V106I	yes	DOR (IR)<br>EFV (LLR)<br>ETR (IR)<br>NVP (IR)<br>RPV (HLR)	Resistance to all NRTIs (except TDF), RPV
29	UKN	B	E138A	no	ETR (PLLR)<br>RPV (LLR)	Resistance to RPV
30	MSM	A1	K103KN, E138A	yes	EFV (HLR)<br>ETR (PLLR)<br>NVP (HLR)<br>RPV (LLR)	Resistance to EFV, NVP RPV

MSM-Men who have sex with men, DOR-Doravirine, EFV-Efavirenz, ETR-Etravirine, NVP-Nevirapine, RPV-Rilpivirine, TDF-Tenofovir, NRTI-nucleoside reverse transcriptase inhibitors, PLLR-Potential low-level resistance, LLR-Low-level resistance, IR-Intermidiate resistance, HLR-High-level resistance

Table S6. Comparison of selected parameters of newly diagnosed people living with HIV-1 (PLHIV) included in the study period 2019-2022 with SDRMs and without SDRMs

Persons	All, n=270, (%)	Persons with SDRM	Persons without SDRM	p-value
**Gender**[a]		45 (16.7)	225 (83.3)	0.718
Male	254 (94.1)	42 (93.3)	212 (94.2)	
Female	16 (5.9)	3 (6.8)	13 (5.8)	
**Transmission risk**[b]				0.356
MSM	231 (85.6)	38 (84.4)	193 (85.8)	
Heterosexual	32 (11.9)	6 (13.3)	26 (11.6)	
IDU*	1 (0.4)	/	1 (0.4)	
Unknown*	6 (2.2)	1 (2.2)	5 (2.2)	
**Age at HIV diagnosis, median years (Q1–Q3)**[c]	38.0 (29.1-46.6)	33.1 (27.8-48.7)	38.6 (29.5-46.5)	0.356
**Stage at HIV diagnosis**[a]				0.888
Acute infection	114 (42.2)	20 (44.4)	94 (41.8)	
Chronic infection (late presenters)	51 (18.9)	7 (15.6)	44 (19.6)	

Chronic infection (very late presenters)	104 (38.5)	17 (37.8)	87 (38.7)	
Unknown*	1 (0.4)	1 (2.2)	/	
**HIV subtype, n (%)[b]**				0.148
B	198 (73.3)	36 (80.0)	162 (72.0)	
non-B/recombinants	72 (26.7)	9 (20.0)	63 (28.0)	
**Baseline CD4+T cells/uL, median (Q1-Q3)[c]**	284.0 (95.0-460.0)	329.5 (91.0-492.0)	277.0 (102.0-450.5)	0.733
**Log10 baseline plasma viraemia, median (Q1-Q3)[c]**	5.0 (4.4-5.5)	4.8 (4.4-5.4)	5.1 (4.5-5.6)	0.214

SDRMs:Surveillance drug resistance mutations; MSM: men who have sex with men; IDU: injecting drug users; Q1, Q3: first and third quartile; n: number of individuals; CD4+ was unavailable for one person;a: associations for categorical variables were tested using Chi-squared test; b: associations for categorical variables were tested using Fisher’s exact test; c: associations for continuous variables were tested using Mann-Whitney Wilcoxon test; *variables with small number of samples were excluded from the sum of proportions and the statistical analysis.

Table S7. Comparison of SDRMs detected by Sanger sequencing (SS) and deep sequencing (DS)

SAMPLE	Accession numbers (RTPR/IN)	Accession number DS	MUTATION CONCORDANCE	SS SDRM	DS SDRM	Frequency of SDRM by DS, n (%)	DS Coverage, number of reads
H1	OR606136/OR605955	E-MTAB-13563	DISCORDANT	L100I, K103KN	T215S	7.41%	1349
H2	OR606174/OR605784	E-MTAB-13563	PARTIAL	K101E	K101E<br>T215S	51.2%<br>12.05%	4518<br>1543
H3	OR613872/OR605972	E-MTAB-13563	PARTIAL	V32I,<br>I47V,<br>F53L,<br>L100I,<br>K103N,<br>T215D	I50V,<br>L100I,<br>K103N,<br>T215D,<br>T215S	5.07%,<br>23.35%<br>27.5%<br>7.03%<br>7.68%	750<br>1219<br>1262<br>2474<br>2474
H4	OR606267/OR605876	E-MTAB-13563	DISCORDANT	/	T215S	7.97%	16424
H5	OR606081/OR605867	E-MTAB-13563	DISCORDANT	/	T215S	18.34%	6740
H6	OR606041/OR605794	E-MTAB-13563	PARTIAL	L210W, T215S	T215S	7.07%	2517
H7	OR606080/OR605865	E-MTAB-13563	COMPLETE	L210W, T215S	L210W<br>T215S	14.67%<br>57.51%	1841<br>1845
H8	OR606024/OR605778	E-MTAB-13563	COMPLETE	T215S	T215S	48.12%	18861
H9	OR606175/OR605775	E-MTAB-13563	PARTIAL	K101E	K101E<br>T215S	59.82%<br>5.63%	6606<br>4599
H10	OR606135/OR605799	E-MTAB-13563	DISCORDANT	/	T215S	5.14%	2490
H11	OR606231/OR605855	E-MTAB-13563	PARTIAL	K103N, P225H	K103N<br>T215S<br>P225H	8.09%<br>10.66%<br>11.02%	742<br>10750<br>10849
H12	OR606128/OR605900	E-MTAB-13563	DISCORDANT	/	T215S	22.11%	1624
H13	OR606133/OR605770	E-MTAB-13563	DISCORDANT	/	T215S	8.59%	3491

H14	OR606047/OR605949	E-MTAB-13563	DISCORDANT	/	T215S	16.86%	5723
H15	OR606083/OR605884	E-MTAB-13563	COMPLETE	T215S	T215S	10.85%	3861
H16	OR606213/OR605932	E-MTAB-13563	COMPLETE(NO SDRM)	/	/	/	/
H17	OR606253/OR605877	E-MTAB-13563	DISCORDANT	K219R	K101E<br>T215S	48.4%<br>5.98%	1903<br>3445
H18	OR606153/OR605899	E-MTAB-13563	DISCORDANT	/	T215S	8.17%	7973
H19	OR606119/OR605910	E-MTAB-13563	PARTIAL	M41L,<br>T215Y	M41L<br>Q148R<br>T215Y<br>T215S	57.85%<br>66.84%<br>48.92%<br>7.41%	2446<br>187<br>10703<br>10703
H20	OR606185/OR605833	E-MTAB-13563	COMPLETE	T215S	T215S	92.55%	26106
H21	OR606035/OR605879	E-MTAB-13563	COMPLETE	T215S,<br>L210W	T215S<br>L210W	11.59%<br>1.28%	9552<br>9553
H22	OR606172/OR605981	E-MTAB-13563	COMPLETE	T215S	T215S	16.56%	11273
H23	OR606274/OR605821	E-MTAB-13563	DISCORDANT	/	T215S	6.19%	3441
H24	OR606134/OR605804	E-MTAB-13563	DISCORDANT	/	T215S	5.51%	3015
H25	OR606170/OR605995	E-MTAB-13563	DISCORDANT	/	T215S	13.96%	8140
H26	OR606144/OR605901	E-MTAB-13563	DISCORDANT	/	Q148R	6.78%	118
H27	OR606123/ND	E-MTAB-13563	DISCORDANT	/	T215S	11.5%	41474
H28	OR606273/OR605843	E-MTAB-13563	DISCORDANT	/	T215S	20.13%	36749
H29	OR613874/OR605862	E-MTAB-13563	DISCORDANT	/	M184V<br>T215S	6.72%<br>12.12%	28885<br>41210
H30	OR606155/OR605891	E-MTAB-13563	COMPLETE (NO SDRM)	/	/	/	/

H31	OR606240/OR605936	E-MTAB-13563	DISCORDANT	/	T215S	14.56%	3126
H32	OR606068/OR605930	E-MTAB-13563	DISCORDANT	/	T215S	11.62%	9012
H33	ORO606052/OR605783	E-MTAB-13563	DISCORDANT	/	T215S	8.2%	16132
H34	OR606276/OR605909	E-MTAB-13563	DISCORDANT	/	T215S	14.4%	6328
H35	OR606224/OR605878	E-MTAB-13563	COMPLETE	T215S	T215S	33.42%	9174
H36	OR606214/OR605886	E-MTAB-13563	PARTIAL	K101E	K101E T215S	72.62% 11.74%	3842 4470
H37	OR606164/OR605774	E-MTAB-13563	COMPLETE	T215S	T215S	18.56%	6321
H38	OR606232/OR605819	E-MTAB-13563	DISCORDANT	/	T215S	15.93%	5563
H39	OR606131/OR605785	E-MTAB-13563	DISCORDANT	/	T215S	8.73%	5902
H40	OR606057/OR605912	E-MTAB-13563	COMPLETE (NO SDRM)	/	/	/	/
H41	OR606261/OR605759	E-MTAB-13563	COMPLETE (NO SDRM)	/	/	/	/
H42	OR606275/OR605923	E-MTAB-13563	DISCORDANT	/	T215S	13.93%	3138
H43	OR606208/OR605842	E-MTAB-13563	COMPLETE (NO SDRM)	/	/	/	/
H44	OR606259/OR605887	E-MTAB-13563	DISCORDANT	/	M41L	8.45%	3797
H45	OR606148/OR605916	E-MTAB-13563	DISCORDANT	/	T215S	13.2%	12399
H46	OR606193/OR606000	E-MTAB-13563	DISCORDANT	/	T215S	12.46%	7158
H47	OR606027/OR605857	E-MTAB-13563	DISCORDANT	/	T215S	11.77%	5402
H48	OR606109/OR605983	E-MTAB-13563	COMPLETE (NO SDRM)	/	/	/	/

H49	OR606126/OR605968	E-MTAB-13563	DISCORDANT	/	T215S	18.63%	1020
H50	OR606023/OR605988	E-MTAB-13563	COMPLETE	T215S	T215S	89.62%	3785
H51	OR606143/OR605793	E-MTAB-13563	DISCORDANT	/	N155S<br>M184V<br>T215S	10.95%<br>13.2%<br>11.81%	493<br>12398<br>18557
H52	OR606066/OR605764	E-MTAB-13563	DISCORDANT	/	T215S	7.78%	5680
H53	OR606241/OR605817	E-MTAB-13563	DISCORDANT	/	K65R<br>T215S	5.08%<br>13.79%	67867<br>5894
H54	OR606160/OR605777	E-MTAB-13563	DISCORDANT	K103N	M184V<br>T215S	30.45%<br>13.67%	11756<br>14730
H55	OR606111/OR605975	E-MTAB-13563	DISCORDANT	/	T215S	7.46%	24610
H56	OR613875/ND	E-MTAB-13563	DISCORDANT	/	M184V<br>T215S	28.72%<br>5.41%	27413<br>46398
H57	OR606043/OR605846	E-MTAB-13563	COMPLETE	T215S	T215S	78.0%	19792
H58	OR606142/OR605971	E-MTAB-13563	COMPLETE (NO SDRM)	/	/	/	/
H59	OR606029/OR605859	E-MTAB-13563	COMPLETE (NO SDRM)	/	/	/	/
H60	OR606168/OR605813	E-MTAB-13563	COMPLETE (NO SDRM)	/	/	/	/

SDRMs:Surveillance drug resistance mutations; SS-Sanger sequencing; DS-deep sequencing; ND-not determined

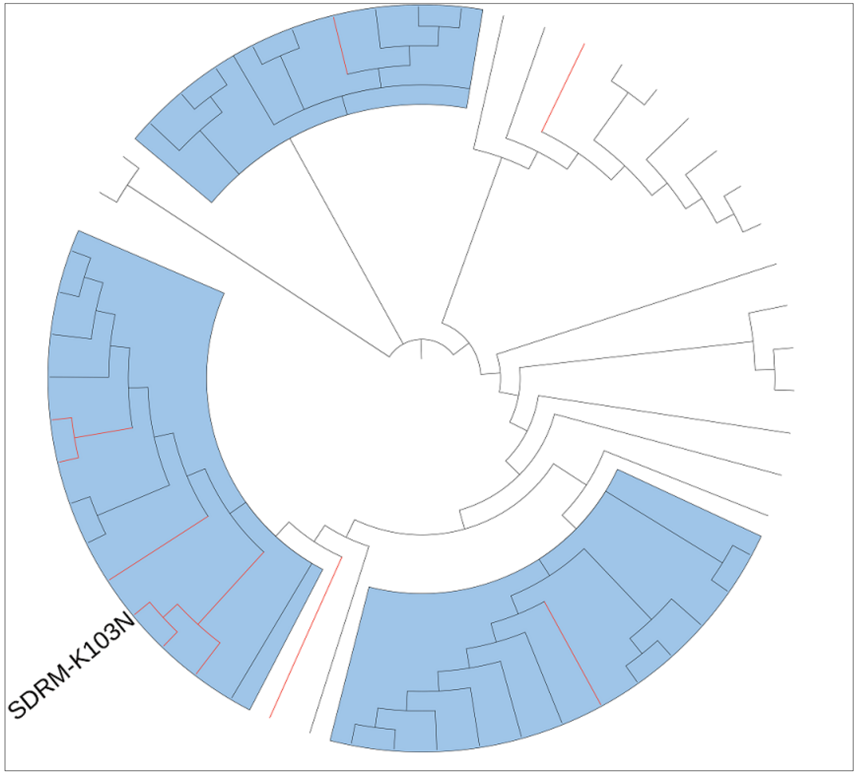

Figure S2. Maximum likelihood phylogenetic tree of the Croatian HIV-1 sequences subtype A1 and corresponding background sequences Branches of Croatian sequences are colored red and all background sequences are colored black. Sequences carrying surveillance drug resistance mutation (SDRM) are marked on the tree. TCs with <75% of Croatian sequences (mixed clusters) are highlighted light blue.

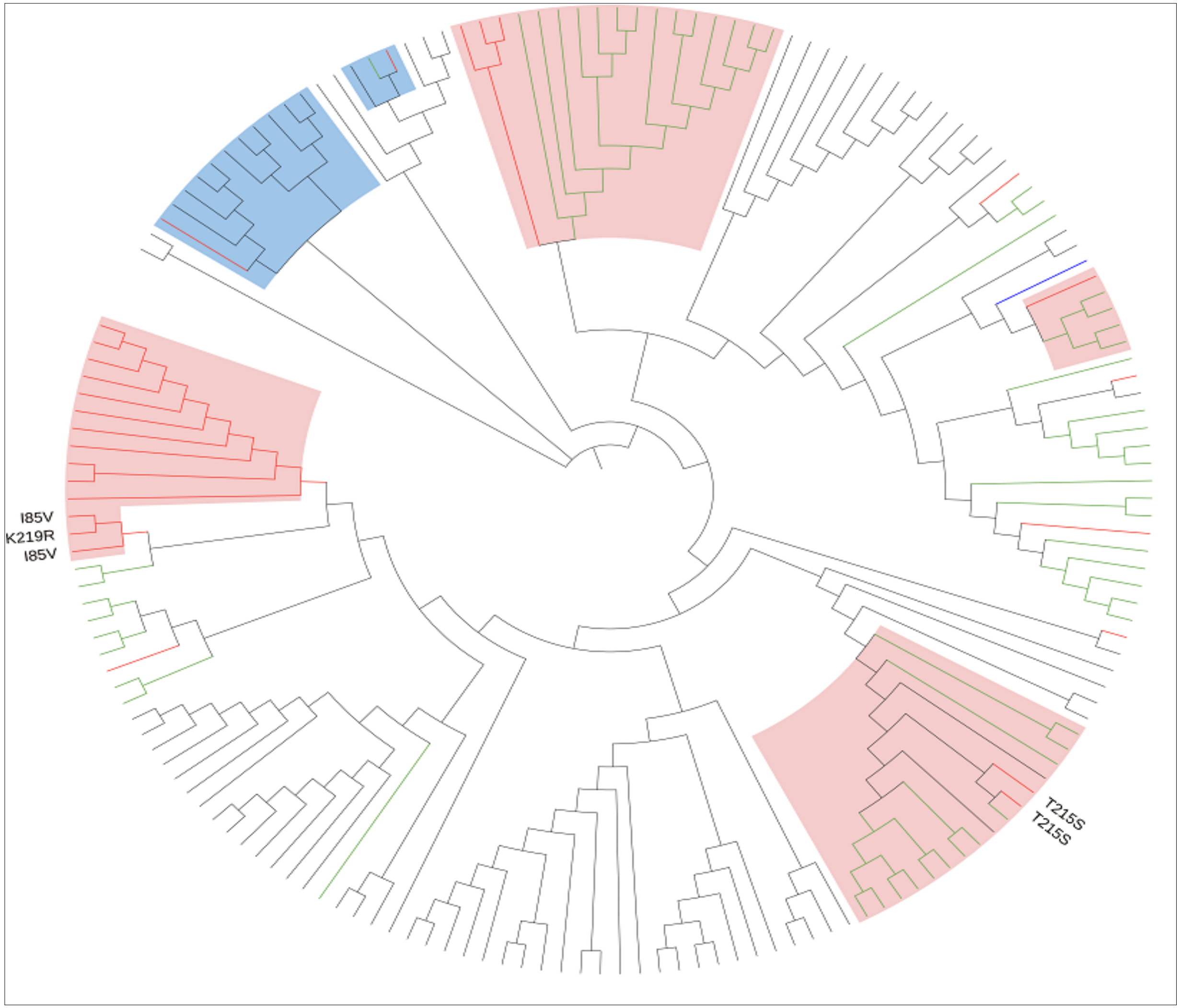
I85V
K219R
I85V
T215S
T215S

Figure S3. Maximum likelihood phylogenetic tree of the Croatian HIV-1 B-D recombinant sequences and corresponding background sequences. For all Croatian sequences (new dataset) transmission risk is marked on the tree: MSM, men who have sex with men-red; HETERO, heterosexual-blue. Croatian sequences (old dataset) are colored green. Sequences carrying surveillance drug resistance mutation (SDRM) are marked on the tree. TCs with >75% of Croatian sequences (local clusters) are highlighted light pink, while TCs with <75% of Croatian sequences (mixed clusters) are highlighted light blue.
